# A Case Study of Depression in High Achieving Students Associated With Moral Incongruence, Spiritual Distress, and Feelings of Guilt

**DOI:** 10.7759/cureus.36102

**Published:** 2023-03-13

**Authors:** Bahjat Najeeb, Muhammad Faisal Amir Malik, Asad T Nizami, Sadia Yasir

**Affiliations:** 1 Institute of Psychiatry, Rawalpindi Medical University, Rawalpindi, PAK

**Keywords:** mutism, major depressive disorder, spiritual distress, moral incongruence, guilt, depression

## Abstract

Psychosocial and cultural factors play an important, but often neglected, role in depression in young individuals. In this article, we present two cases of young, educated males with major depressive disorder and prominent themes of guilt and spiritual distress. We explore the relationship between moral incongruence, spiritual distress, and feelings of guilt with major depressive episodes by presenting two cases of depression in young individuals who were high-achieving students. Both cases presented with low mood, psychomotor slowing, and selective mutism. Upon detailed history, spiritual distress and feelings of guilt due to internet pornographic use (IPU) and the resulting self-perceived addiction and moral incongruence were linked to the initiation and progression of major depressive episodes. The severity of the depressive episode was measured using the Hamilton Depression Scale (HAM-D). Themes of guilt and shame were measured using the State of Guilt and Shame Scale (SSGS). High expectations from the family were also a source of stress. Hence, it is important to keep these factors in mind while managing mental health problems in young individuals. Late adolescence and early adulthood are periods of high stress and vulnerabe to mental illness. Psychosocial determinants of depression in this age group generally go unexplored and unaddressed leading to suboptimal treatment, particularly in developing countries. Further research is needed to assess the importance of these factors and to determine ways to mitigate them.

## Introduction

More attention needs to be paid to the psychological and societal factors which precipitate, prolong, and cause a relapse of depression in high-achieving young individuals. A young, bright individual has to contend with the pressures of -- often quite strenuous -- moral and financial expectations from the family, moral incongruence, spiritual distress, and feelings of guilt.

Moral incongruence is the distress that develops when a person continues to behave in a manner that is at odds with their beliefs. It may be associated with self-perceptions of addictions, including, for example, to pornographic viewing, social networking, and online gaming [1]. Perceived addiction to pornographic use rather than use is related to the high incidence of feelings of guilt and shame and predicts religious and spiritual struggle [2-3]. Guilt is a negative emotional and cognitive experience that occurs when a person believes that they have negated a standard of conduct or morals. It is a part of the diagnostic criteria for depression and various rating scales for depressive disorders [4]. Generalized guilt has a direct relationship with major depressive episodes. Guilt can be a possible target for preventive as well as therapeutic interventions in patients who experience major depressive episodes [5].

We explored the relationship between moral incongruence, spiritual distress, and feelings of guilt with major depressive episodes in high-achieving students. Both patients presented with symptoms of low mood, extreme psychomotor slowing, decreased oral intake, decreased sleep, and mutism. The medical evaluation and lab results were unremarkable. The severity of depressive episodes was measured using the Hamilton Depression Scale (HAM-D). Themes of guilt and shame were measured by using the State of Guilt and Shame Scale (SSGS). This case study was presented as a poster abstract at the ‘RCPsych Faculty of General Adult Psychiatry Annual Conference 2021.’

## Case presentation

Case 1

A 25-year-old Sunni Muslim, Punjabi male educated till Bachelors presented with a one-month history of fearfulness, weeping spells during prolonged prostration, social withdrawal, complaints of progressively decreasing verbal communication to the extent of giving nods and one-word answers, and decreased oral intake. His family believed that the patient's symptoms were the result of ‘Djinn’ possession. This was the patient’s second episode. The first episode was a year ago with similar symptoms of lesser severity that resolved on its own. Before being brought to us, he had been taken to multiple faith healers. No history of substance use was reported. Psychosexual history could not be explored at the time of admission. His pre-morbid personality was significant for anxious and avoidant traits. 

On mental state examination (MSE), the patient had psychomotor retardation. He responded non-verbally, and that too slowly. Once, he wept excessively and said that he feels guilt over some grave sin. He refused to explain further, saying only that ‘I am afraid of myself.’ All baseline investigations returned normal. His score on the Hamilton Depression Rating Scale (HAM-D) was 28 (Very Severe). A diagnosis of major depressive disorder was made. The patient was started on tab sertraline 50 mg per day and tab olanzapine 5 mg per day. After the second electroconvulsive therapy (ECT), his psychomotor retardation improved and he began to open up about his stressors. His HAM-D score at this time was 17 (moderate). He revealed distress due to feelings of excessive guilt and shame due to moral incongruence secondary to internet pornography use (IPU). The frequency and duration of IPU increased during the last six months preceding current illness. That, according to him, led him to withdraw socially and be fearful. He felt the burden of the high financial and moral expectations of the family. He complained that his parents were overbearing and overinvolved in his life. His family lacked insight into the cause of his illness and had difficulty accepting his current state. All these factors, particularly spiritual distress, were important in precipitating his illness. He scored high on both the shame and guilt domains (14/25, and 20/25, respectively) of the State of Shame and Guilt Scale (SSGS).

He was discharged after three weeks following a cycle of four ECTs, psychotherapy, and psychoeducation of the patient and family. At the time of discharge, his HAM-D score was 10 (mild) and he reported no distress due to guilt or feeling of shame. He has been doing well for the past 5 months on outpatient follow-up.

Case 2

A 21-year-old Sunni Muslim, Punjabi male, high-achieving student of high school presented with low mood, low energy, anhedonia, weeping spells, decreased oral intake, decreased talk, and impaired biological functions. His illness was insidious in onset and progressively worsened over the last 4 months. This was his first episode. He was brought to a psychiatric facility after being taken to multiple faith healers. Positive findings on the MSE included psychomotor slowing, and while he followed commands, he remained mute throughout the interview. Neurological examination and laboratory findings were normal. His score on HAM-D was 24 (very severe). He was diagnosed with major depressive disorder and started on tab lorazepam 1 mg twice daily with tab mirtazapine 15 mg which was built up to 30 mg once daily. He steadily improved, and two weeks later his score on HAM-D was 17 (moderate). His scores on SSGS signified excessive shame and guilt (16/25, and 21/25; respectively). He communicated his stressors which pertained to the psychosexual domain: he started masturbating at the age of 15, and he felt guilt following that but continued to do so putting him in a state of moral incongruence. He perceived his IPU as ‘an addiction’ and considered it a ‘gunahe kabira’ (major sin) and reported increased IPU in the months leading to the current depressive episode leading to him feeling guilt and anguish. Initially, during his illness, he was taken to multiple faith healers as the family struggled to recognize the true nature of the disease. Their understanding of the illness was of him being under the influence of ‘Kala Jadu’ (black magic). His parents had high expectations of him due to him being their only male child. After 3 weeks of treatment and psychotherapy, his condition improved and his HAM-D score came out to be 08 (mild). He was discharged on 30 mg mirtazapine HS and seen on fortnightly visits. His guilt and shame resolved with time after the resolution of depressive symptoms and counseling. We lost the follow-up after 6 months.

## Discussion

Late adolescence and young adulthood can be considered a unique and distinct period in the development of an individual [6]. It is a period of transition marked by new opportunities for development, growth, and evolution, as well as bringing new freedom and responsibilities. At the same time, this period brings interpersonal conflicts and an increased vulnerability to mental health disorders such as depression and suicidality. Biological, social, and psychological factors should all be explored in the etiology of mental health problems presenting at this age [6].

Socio-cultural factors played a significant role in the development and course of disease in our patients, and these included the authoritarian parenting style, initial lack of awareness about psychiatric illnesses causing a delay in seeking treatment, high expressed emotions in the family, and the burden of expectations from the family and the peer group. The strict and often quite unreasonable societal and family expectations in terms of what to achieve and how to behave and the resultant onus on a high-scoring, bright young individual make for a highly stressful mental state. 

We used the ICD-10 criteria to diagnose depression clinically in our patients and the HAMD-17 to measure the severity of symptoms [7]. Both our patients had scores signifying severe depression initially. Psychomotor retardation was a prominent and shared clinical feature. Psychomotor retardation is the slowing of cognitive and motor functioning, as seen in slowed speech, thought processes, and motor movements [8-9]. The prevalence of psychomotor retardation in major depressive disorder ranges from 60% to 70% [10]. While psychomotor retardation often responds poorly to selective serotonin reuptake inhibitors (SSRI), both tricyclic antidepressants (TCAs) and noradrenergic and specific serotonergic antidepressants (NaSSA) produce a better response [9, 11]. In addition, ECT shows a high treatment response in cases with significant psychomotor retardation [11-12].

A growing body of evidence shows that shame and guilt are features of numerous mental health problems. Guilt is the negative emotional and cognitive experience that follows the perception of negating or repudiating a set of deeply held morals [4]. Guilt can be generalized as well as contextual and is distinct from shame [13]. The distinction between guilt and shame allows for an independent assessment of the association of both guilt and shame with depressive disorder. As an example, a meta-analysis of 108 studies including 22,411 individuals measuring the association of shame and guilt in patients with depressive disorder found both shame and guilt to have a positive association with depressive symptoms. This association was stronger for shame (r=0.43) than for guilt (r=0.28) [14]. In our study, we used the State of Shame and Guilt Scale (SSGS), to measure the feelings of guilt and shame [15]. The SSGS is a self-reported measure and consists of 5 items each for subsets of guilt and shame. SSGS scores showed high levels of guilt and shame in both of our patients.

During the course of treatment, we paid special attention to the psychological, cultural, and social factors that likely contributed to the genesis of the illness, delayed presentation to seek professional help, and could explain the recurrence of the depressive episodes. In particular, we observe the importance, particularly in this age group, of family and societal pressure, spiritual distress, moral incongruence, and feelings of guilt and shame. Moral incongruence is when a person feels that his behavior and his values or judgments about that behavior are not aligned. It can cause a person to more negatively perceive a behavior. As an example, the presence of moral congruence in an individual is a stronger contributor to perceiving internet pornographic use (IPU) as addictive than the actual use itself [16]. Therefore, moral congruence has a significant association with increased distress about IPU, enhanced psychological distress in general, and a greater incidence of perceived addiction to IPU [16].

Self-perceived addiction is an individual’s self-judgment that he or she belongs to the group of addicts. The pornography problems due to moral incongruence (PPMI) model is one framework that predicts the factors linking problematic pornographic use with self-perceived addiction. This model associates moral incongruence with self-perceived addiction to problematic pornographic use [17]. A recent study on the US adult population also showed a high association of guilt and shame with moral incongruence regarding IPU [18]. Another factor of importance in our patients was spiritual distress, which is the internal strain, tension, and conflict with what people hold sacred [19]. Spiritual distress can be intrapersonal, interpersonal, or supernatural [20]. Research indicates that IPU causes people to develop spiritual distress that can ultimately lead to depression [16-17].

## Conclusions

In both our cases the initial presentation was that of psychomotor slowing, selective mutism, and affective symptoms of low mood, therefore, a diagnosis of depressive illness was made. One week into treatment, improvement was noted both clinically as well as on the psychometric scales. Upon engaging the patients to give an elaborate psychosexual history, moral incongruence, spiritual distress, and feelings of guilt, linked particularly to self-perceived addiction to IPU were found. Sensitivity to the expectations of the parents, the cognizance of failing them because of illness, and their own and family’s lack of understanding of the situation were additional sources of stress. Hence, it is imperative to note how these factors play an important role in the initiation, progression, and relapse of mental health problems in young individuals. 
